# Comparing the Sexual Reproductive Success of Two Exotic Trees Invading Spanish Riparian Forests vs. a Native Reference

**DOI:** 10.1371/journal.pone.0160831

**Published:** 2016-08-16

**Authors:** Isabel Cabra-Rivas, Pilar Castro-Díez

**Affiliations:** Departamento de Ciencias de la Vida, Unidad Docente de Ecología, Facultad de Biología, Ciencias Ambientales y Química, Universidad de Alcalá, N-II, Km 33.6, PO Box 20, Alcalá de Henares (28805), Madrid, Spain; Universidade da Coruna, SPAIN

## Abstract

A widely accepted hypothesis in invasion ecology is that invasive species have higher survival through the early stages of establishment than do non-invasive species. In this study we explore the hypothesis that the sexual reproductive success of the invasive trees *Ailanthus altissima* (Mill.) Swingle and *Robinia pseudoacacia* L. is higher than that of the native *Fraxinus angustifolia* Vahl., all three species coexisting within the riparian forests of Central Spain. We compared different stages of the early life cycle, namely seed rain, seed infestation by insects, seed removal by local fauna, seed germination under optimal conditions and seedling abundance between the two invasive trees and the native, in order to assess their sexual reproductive success. The exotic species did not differ from the native reference (all three species displaying high seed rain and undergoing seed losses up to 50% due to seed removal by the local fauna). Even if the exotic *R*. *pseudoacacia* showed a high percentage of empty and insect-parasited seeds along with a low seedling emergence and the exotic *A*. *altissima* was the species with more viable seeds and of higher germinability, no differences were found regarding these variables when comparing them with the native *F*. *angustifolia*. Unsuitable conditions might have hampered either seedling emergence and survival, as seedling abundance in the field was lower than expected in all species -especially in *R*. *pseudoacacia*-. Our results rather suggest that the sexual reproductive success was not higher in the exotic trees than in the native reference, but studies focusing on long-term recruitment would help to shed light on this issue.

## Introduction

Invasions by non-native plant species are a considerable and costly threat to diversity and ecosystem functioning worldwide [1, 2]. Since Baker’s description of the successful invader [3], many authors have tried to identify traits that could make invaders more successful than native species [4, 5, 6, 7]. Many of these traits are related to a prolific and successful sexual reproduction such as reproduction at an early age, high fecundity, extended flowering period or long-distance dispersal [8, 5, 9]. Also the ability for vegetative reproduction, particularly the ability to resprout, is considered as a part of the “invader syndrome” [10, 5], as it contributes to a rapid local dominance. However, sexual reproduction provides the species with a higher potential for rapid local adaptation [11, 12], whereas vegetative reproduction may lead to the depletion of genotypic variability within clonal populations over time, making them more susceptible to diseases, pathogens, and environmental stochasticity [13]. Additionally, sexual reproduction potentially contributes to longer-distance dispersal than vegetative reproduction [14, 15]. These facts highlight the relevance of sexual reproduction for an exotic plant species to overcome several of the barriers to become a successful invader in a new area, specifically, establishment of self-sustainable healthy populations and fast spread from the original point of introduction [16, 17].

**Fig 1:**
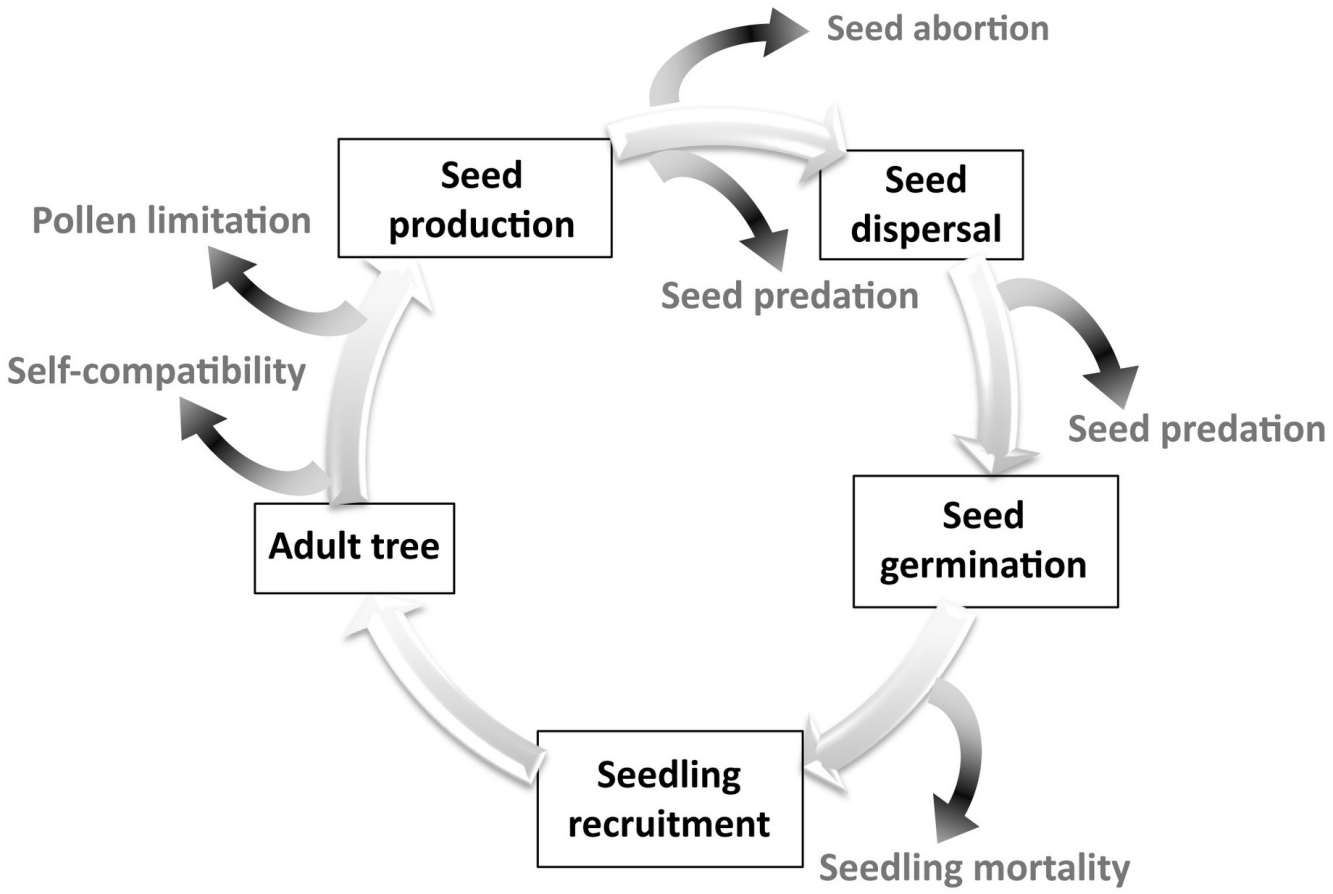
Stages of the sexual reproductive cycle of a plant. Seed losses at early stages of the plant cycle are represented in dark grey.

For sexual reproduction to become successful, different processes need to get linked through time: effective flower pollination, production of enough quantities of viable seeds, a fraction of which must be able to germinate, and finally, seedlings must attain a successful recruitment. Along this process, losses may occur at each step, such as production of empty seeds (i.e parthenocarpy, embryo abortion), seed predation -before or after dispersal-, germination failure, or seedling mortality (Fig 1). Indeed, it is at the early stages of the plant cycle that plants experience the highest mortality rates of any life history stage [18].

It is commonly accepted that invasive species have a higher survival through the early stages of establishment than do non-invasive species [4, 19, 5]. For instance, herbivores or granivores may prefer seeds from native over non-native species [20, 21, 22], as suggested by the “Enemy Release Hypothesis” [23, 24]. Additionally, invasive species have been often found to have higher germination rates and seedling survival when compared with natives [5, 25]. Thus, assessing the success of the different steps leading to a successful sexual reproduction of invasive species (i.e. seed production, seed predation, germination and recruitment) may help to identify the most and the least vulnerable steps, which may be of interest for a cost-effective management of invasions [26, 5, 27, 28]. Yet, little work has simultaneously examined all these steps directly comparing coexisting invasive and native species.

Riparian forests are highly susceptible to invasion by alien plant species due to the frequent natural and human-induced disturbances they are exposed to, their high water and nutrient availability and the water-aided dispersal of propagules [29, 30]. In fact, this type of habitat is one of the most invaded in Europe, and particularly in continental Mediterranean regions [31]. *Ailanthus altissima* and *Robinia pseudoacacia* are two exotic trees which have efficiently colonized riparian forests of Central Spain and their populations seem to be expanding [32].

Our general aim with this study is to compare the sexual reproductive success between two invasive trees (*A*. *altissima* and *R*. *pseudoacacia*) spreading through riparian forests of Central Spain and the coexisting native tree *Fraxinus angustifolia*. Particularly, we aim to assess their differences across four stages affecting the final regeneration process: 1) seed rain, 2) seed predation, 3) seed germination and 4) seedling establishment. We hypothesize that invaders will show the highest sexual reproductive success either due to a greater seed rain [33, 34], a lower seed predation, a higher germination rate, and/or a higher seedling establishment [5, 9, 35, 25].

## Materials and Methods

Field permit was granted by: Desprosa S.A. (El Encín) and Rosendo Elvira (Jardín Botánico de la Universidad de Alcalá)

### Study site

The study was carried out in the floodplain of the middle Henares River (Tagus Basin), Central Spain (Madrid province, 40°29’N, 3°19’W). The climate is continental Mediterranean with cold winters and hot and dry summers. Mean annual temperature is 13.5°C and mean annual precipitation is 358 mm (weather station of Alcalá de Henares-El Encín, 600 m.a.s.l., period 1970–2009). Native communities are dominated by *Populus alba* L., *Salix alba* L., *Populus nigra* L., *Fraxinus angustifolia* Vahl. and *Ulmus minor* Mill. [36, 37]. However, during the last decades *A*. *altissima* and *R*. *pseudoacacia* have spread through [38, 39].

### Study species

*Ailanthus altissima* (Mill.) Swingle (Simaroubaceae), native to SE Asia, and *R*. *pseudoacacia* L. (Fabaceae), native to North America, are considered global invaders, being among the 100 worst invasive species in Europe and among the 20 most harmful in Spain [40, 41, 42]. Both species were introduced during the 18^th^ century in Spain mostly for ornamental purposes. They occupy disturbed areas as well as (semi-)natural habitats [32, 43, 44]. They ripe fruits between June-October (*A*. *altissima*) and May-September (*R*. *pseudoacacia*) [45, 46], producing abundant seed yields [47, 48] dispersed all over the year. *Ailanthus altissima* is a wind-dispersed dioecious species [49], whereas *R*. *pseudoacacia* is monoecious [50] and produces barochorous dormant seeds also dispersed by birds [51, 52]. The native *F*. *angustifolia* Vahl. (Oleaceae) was selected as a native reference because it often coexists with the invaders in the outer vegetation strip of riparian forests and is the only native tree that shares several functional traits with them (i.e. compound leaves, similar reproductive phenology, and seed mass). It is a dioecious wind-dispersed tree species that completes fruit set in September-October [53].

### Study plots

Along an 80 km stretch of the middle Henares River we selected patches of forests dominated by three different tree species: *A*. *altissima*, *R*. *pseudoacacia* or *F*. *angustifolia*, occurring under similar environmental conditions. For each of the three selected forest types, we delimited three 300 m^2^ independent plots (nine plots in total, separated at least 50 m apart from each other) based on their proximity to the river (<100 m, Table 1) and the dominance of each target species. The diameter at breast height (*dbh*) of every adult tree (*dbh* ≥7 cm or height > 1.3 m) was recorded in every plot in order to calculate the basal area (*BA* = *dbh*^2^ * π/4) of every adult tree. We only chose those plots where the basal area of the stems of the target species (*BA*_*D*_) represented > 50% of the total basal area including the stems from all species in the stand (*BA*_*T*_); this means, plots where the target species was dominant. Unlike *R*. *pseudoacacia*, *A*. *altissima* tends to form monospecific stands in the study area, however all three species coexist in close distance and even co-occurred in some of the plots (*e*.*g*. *R*. *pseudoacacia* and *F*. *angustifolia*, but see [54] for further details on the community structure and composition in every plot). All plots showed a similar stand structure, with a clear dominance of relatively thin trees and a low frequency of old trees (see S1 Fig). Canopy openness was assessed as the proportion of the area not obstructed by foliage, branches or stems in 5–6 hemispherical photographs [55, 56] per plot taken in September 2012 (Nikon FC-E9 hemispherical lens and a Nikon Coolpix 8400 camera) and was analysed using the open source software ImageJ (http://rsbweb.nih.gov/ij/). Canopy openness was used as a proxy for the forest understory light environment [55].

**Table 1:** Canopy openness (mean±SE), percentage of sexual recruits, number of fruiting trees from the dominant species, mean diameter at breast height (*dbh*) of the dominant tree species, *BA*_*DF*_ (basal area of all fruiting trees of the dominant tree species), *BA*_*T*_ (basalarea of all adult trees (*dbh* ≥ 7 cm or height > 1.3 m) from all species in the plot) and annual standardized seed rain of the dominant species, either in terms of seed biomass and seed number shown by year and 300 m^2^ plot of the study species. Values for *A*. *altissima* are female individuals only.

Plot*	Location	Canopy openness (%)	Sexual recruits (%)	No. fruiting trees	*dbh*	*BA*_*DF*_ (m^2^)**	*BA*_*T*_ (m^2^)	Biomass of seeds		No. seeds	
					(cm)			(g ha^-1^ m^-2^ y^-1^)		(ha^-1^ m^-2^ y^-1^)	
								Year 1	Year 2	Year 1	Year 2
AA1	40° 34’ 31” N	40.8±2.0	39.1	4	23.1±11.7	2.0	7.4	11757	31998	70.8 x 10^4^	209.2 x 10^4^
	3° 13’ 46” W										
AA2	40° 34’ 27” N	19.0±3.9	55.2	31	9.8±3.6	2.6	3.5	6881	91293	40.5 x 10^4^	550.4 x 10^4^
	3° 13’ 46” W										
AA3	40° 39’ 53” N	11.4±2.0	99.0	40	14.7±9.2	9.4	12.3	47190	27355	280.8 x 10^4^	167.7 x 10^4^
	3° 10’ 24” W										
RP1	40° 56’ 49” N	22.5±1.9	6.7	26	13.7±5.2	4.4	4.8	20801	40982	84.9 x 10^4^	178.8 x 10^4^
	2° 56’ 16” W										
RP2	40° 56’ 58” N	37.4±0.8	0.0	21	8.0±1.0	1.1	1.4	9111	1099	35.4 x 10^4^	5.1 x 10^4^
	2° 56’ 1” W										
RP3	40° 56’ 48” N	31.2±2.3	10.0	16	18.6±7.5	5.0	7.6	48682	7207	208.1 x 10^4^	29.3 x 10^4^
	2° 56’ 17” W										
FA1	40° 57’ 18” N	31.8±4.1	71.4	15	19.9±12.2	6.3	6.3	12213	16198	112.7 x 10^4^	162.7 x 10^4^
	2° 55’ 20” W										
FA2	40° 57’ 16” N	41.3±1.7	0.0	25	14.1±5.7	4.5	8.0	32362	38590	218.8 x 10^4^	196.1 x 10^4^
	2° 55’ 50” W										
FA3	40° 30’ 57” N	24.1±1.6	86.4	23	9.9±2.7	1.9	3.3	19444	14576	148.5 x 10^4^	121.7 x 10^4^
	3° 18’ 14” W										

*Plot names: AA: plot with dominance of the invasive species *A*. *altissima*; RP: plot with dominance of the invasive species *R*. *pseudoacacia*; FA: plot with dominance of the native species *F*. *angustifolia*.

**Note that unlike *BA*_*D*_, *BA*_*DF*_ represents only the basal area of **fruiting** trees from the dominant species in the plot.

### Studied variables

#### Seed traits

In spring 2013, ripen seeds from the three species were collected from ten different adult trees per species from the study site and pooled together. From this pool, subsamples were taken to study seed traits, seed germination and seed predation. The studied traits, expected to be correlated with seed predation, were seed mass and nutritional content. Chemical analyses were performed on the dispersal units (i.e., pericarp + seed for *A*. *altissima* and *F*. *angustifolia*, and the seed for *R*. *pseudoacacia*). The dry weight was recorded for 100 seeds per species (measured to 0.001 g). Chemical analysis of protein (Kjeldahl method, [57]), fat (ether extraction technique, [57]), starch (polarimetric method, [57]) and fiber content (solubilisation in acid and basic media with incineration, [57]) were performed on three replicates of 30g of diaspores per species.

#### Seed rain

From June 2011 to May 2013 seed rain was monthly collected in 9 seed traps per plot (total surface of 2.13 m^2^ per plot). Three types of seed traps were used: squared ground traps, pot traps and hanging traps (see more details in [54]). Seeds/fruits of the target species were separated from other material, oven-dried (60°C) for 48h, counted and weighted to calculate seed rain as number and biomass of seeds per species. Given that *A*. *altissima* and *F*. *angustifolia* disperse fruits rather than seeds, a subsample of 100 fruits was selected for each one of these two species to establish the relation between seed and fruit (dispersal unit) weight, and this relation was used to transform fruit into seed biomass. Because seed production is dependent upon tree size and abundance [58, 59], we calculated for each species a mean annual standardized seed rain as the number (*SSN*, ha^-1^ m^-2^ y^-1^) and biomass (*SSB*, g ha^-1^ m^-2^ y^-1^) of seeds annually collected per ha relative to the basal area of all reproductive individuals in the plot (*BA*, m^2^).

#### Seed infestation and seed removal

We randomly chose a subsample of 100 seeds per species from the pool and every seed was opened to determine both the percentage of empty (i.e. seeds with no embryo) and infested seeds (i.e. seeds with either insect emergence hole or larvae inside).

Given that seeds of all three species complete fruit set at the beginning of autumn and many seeds overwinter in the canopy (S2 Fig), we assessed pre- and post-dispersal seed predation (i.e. seed removal from the canopy and from the soil, respectively) through removal experiments carried out in ‘removal plots’ (see below) during autumn (2012) and spring (2013) -which are the seasons of high animal foraging rates because of a high food availability in Mediterranean regions [60]- and averaged them to obtain a representative value of seed removal. On the basis of previous literature in the Mediterranean region, we used seed removal as a surrogate for seed predation [61, 62, 63], although some potential predators could have only relocated and not consumed a proportion of the seeds removed (see limitations of this assumption in [64]), and they may even stimulate germination (see below how we dealt with this possibility). To assess pre-dispersal seed predation (i.e., seed removal from the tree canopy), we selected three 100 m-transects in the middle Henares River riparian forest (40° 30’ 47” N, 1° 18’ 06” W), within pure stands of *P*. *alba* to avoid external inputs of seeds from the study species. Thirty three feeders per transect were placed in the canopy of the trees. Feeders consisted of 4 cm height plastic glasses, with drainage holes, with three dispersal units each (one per species; hereafter called seeds) and were fixed to low branches of the trees. Seeds were periodically checked for removal until stabilization (S3 Fig). Simultaneously, to assess post-dispersal seed predation, we delimited four 14x14 m removal plots within the same area. In each removal plot we anchored 50 15cm-wire stakes in the ground at about 2 m from each other, holding three pieces of nylon fishing line each with a seed of each species glued in the extreme. Glue was odourless and tasteless. Glued seeds were periodically checked for removal, until either seed removal stopped or seed removal curve stabilized (see S4 Fig).

#### Potential germination

In spring 2013, ripen seeds of every study species were collected from ten different adult trees per species from the study site and pooled together. We performed a germination test in germination chambers under optimal conditions (12h photoperiod, photon flux density of 496.2±81.5μmol/m^2^/s, temperature 20.5°C; [65]) on a random subsample of 150 seeds (10 seeds x 15 Petri dishes) per species. Given that *R*. *pseudoacacia* seeds present dormancy [66], we assessed germination with one set of untreated seeds and another set of scarified seeds (1 minute in 90°C water + 24h soaked; [67]) to estimate the maximum potential germination. A seed was considered germinated when radicle emergence was observed. Non-germinated seeds were dissected to exclude non-viable ones (i.e. empty or with larvae inside), so that germination was expressed per unit of viable seeds.

#### Potentially recruitable seeds

We calculated the percentage of potentially recruitable seeds (*PRS*) by subtracting from the initial seed rain the percentage of empty, infested, and viable post-dispersal removed seeds (pre-dispersal removed seeds are not part of the seed rain), and the percentage of seeds that, although putatively viable, would not germinate (based on potential germination tests).

In the case of *R*. *pseudoacacia*, the passage of the seeds through the digestive tract of animals (i.e. birds) may break seed dormancy and enhance germination [68]. Therefore we express *PRS* for *R*. *pseudoacacia* as a range between a minimum (assuming that seed consumption destroy seeds and the viable remaining seeds germinated at the rate of untreated seeds) and a maximum (assuming that all removed seeds were consumed by birds and had the germination rate of scarified seeds).

#### Seedling abundance

In May 2012 we measured the abundance of recruits (plants with *dbh* < 7 cm or height < 1.3 m) in the same plots where we assessed seed rain. In each plot we randomly established three 10 x 2 m transects (covering 60 m^2^ of the 300 m^2^ plot). In every transect the total number of recruits of the target species was counted in five 2 x 2 m quadrats. We randomly chose two recruits per quadrat and excavated them to distinguish seedlings from root suckers. The total amount of recruits was multiplied by the proportion of seedlings and expressed per hectare and per square meter of *BA*_*D*_ to obtain seedling abundance (h^-1^ m^-2^).

### Data analyses

Mean annual seed rain was compared across species and years by means of three-way ANOVA, with species, year and plot as fixed factors. Seed traits (mass, % of proteins, fat, starch and fiber content), percentage of seeds lost at different stages (% of empty seeds, infested seeds -with larvae inside-, pre-dispersal removed seeds, post-dispersal removed seeds), potential germination (%), seedling abundance and the percentage of sexual recruits were compared among species by means of a one-way ANOVA followed by post-hoc Tukey test or with Kruskal-Wallis tests followed by Kruskal-Nemenyi post-hoc tests when parametric assumptions were not met. The potential germination was compared separately for scarified and non-scarified *R*. *pseudoacacia* seeds. Statistical results were corrected after Bonferroni. All the analyses were performed with R 3.0.3 [69].

## Results

### Seed traits

*A*. *altissima* and *R*. *pseudoacacia* showed a similar seed mass and lower than *Fraxinus angustifolia* seeds. *R*. *pseudoacacia* was the species with the highest starch and protein seed content, and the lowest fiber and fat content. *Fraxinus angustifolia* had the lowest protein seed content and *A*. *altissima*, the highest fiber and fat content (Table 2).

**Table 2:** Morphological and chemical seed traits (mean±SE) of the species *A*. *altissima*, *R*. *pseudoacacia* and *F*. *angustifolia*.

Dependent variables	*A*. *altissima* (IN)	*R*. *pseudoacacia* (IN)	*F*. *angustifolia* (NA)	Test
Seed mass (g/seed)	0.017±0.000 ^a^	0.018±0.000 ^a^	0.033±0.001 ^b^	KW
Protein content (%)	15.6±0.1 ^a^	42.5±0.9 ^b^	12.5±0.4 ^c^	AN
Starch content (%)	0 ^a^	8.2±0.9 ^b^	0 ^a^	KW
Fat content (%)	17.3±0.6 ^a^	8.4±0.5 ^b^	11.1±0.2 ^c^	AN
Fiber content (%)	31.4±0.2 ^a^	19.9±0.3 ^b^	30.5±0.1 ^c^	AN

(IN) = invasive; (NA) = native

Different lower case letters indicate significant differences among species based on an ANOVA (AN) /Kruskal-Wallis (KW) followed by post-hoc tests with Bonferroni correction (*P* < 0.02).

### Seed rain, seed infestation and seed removal

Mean annual seed rain (either in terms of seed number or biomass) was not significantly higher in the exotics than in the native tree species (Table 3). It was also similar across years, maybe due to the high interannual variation found during our two-years study (Table 1). However, a trend is observed where invaders are more fecund than the native species. The percentage of viable seeds (not empty, not infested) was 100% in *A*. *altissima*, but only 50% in *R*. *pseudoacacia*, due to a high percentage of empty seeds and to a lesser extent, to parasitic insects (Table 3). In *F*. *angustifolia* 79% of the seeds were viable, 19% were empty and only 2% infested. Seed removal, either in the canopy or in the ground, was high for all species (55–76%) but neither pre- nor post dispersal seed removers showed a marked preference for native or invasive seeds (Table 3).

**Table 3:** Annual standardized seed rain (*SSN*; N = 3), annual standardized seed rain biomass (*SSB*; N = 3), average percentage of pre- (*PRE*; N = 3) and post-dispersal (*POS*; N = 4) removed seeds, percentage of empty seeds (*EMP*; N = 10), percentage of infested seeds (*INF*; N = 10), potential germination (*PG*; N = 15. In *R*. *pseudoacacia* potential germination under scarification is shown in brackets), percentage of potentially recruitable seeds (*PRS*) from the seed rain (and standardized number below), standardized seedling abundance (*SSAB*; N = 3) and percentage of sexual recruits in the species *A*. *altissima*, *R*. *pseudoacacia* and *F*. *angustifolia* from the middle Henares River riparian forest. Values are shown as mean±SE. Variables where significant differences were found among species are shown in bold.

Dependent variables	*A*. *altissima* (IN)	*R*. *pseudoacacia* (IN)	*F*. *angustifolia* (NA)	Test
*SSN* (ha^-1^ m^-2^ y^-1^)	(219.9±75.3) x 10^4 a^	(160.1±16.9) x 10^4 a^	(90.3±34.5) x 10^4 a^	AN
*SSB* (g ha^-1^ m^-2^ y^-1^)	36078±12528 ^a^	22230±4372 ^a^	21313±7942 ^a^	AN
*PRE* (%)	72.2±7.3 ^a^	69.7±3.2 ^a^	55.6±6.2 ^a^	AN
*POS* (%)	60.6±5.4 ^a^	75.9±2.9 ^a^	59.5±9.6 ^a^	AN
***EMP*** (%)	0±0 ^a^	35.0±16.5 ^b^	19.0±14.5 ^a,b^	KW
***INF*** (%)	0±0 ^a^	15.0±13.5 ^b^	2±4.2 ^a,b^	KW
***PG*** (%)	67.0±3.9 ^a^	14.1±3.0 ^b^ (68.2±3.7 ^a^)	14.2±3.6 ^b^	KW
*PRS* (%)	26.4%	1.7–33%	4.4%	-
*PRS* (ha^-1^ m^-2^ y^-1^)	58.5 x 10^4^	2.7 x 10^4^−52.8 x 10^4^	4.0 x 10^4^	-
*SSAB* (ha^-1^ m^-2^)	9000±4525 ^a^	46±27 ^a^	1187±1048 ^a^	KW
Sexual recruits (%)	64.4±17.9 ^a^	5.6±2.9 ^a^	52.6±26.7 ^a^	AN

(IN) = invasive; (NA) = native

Different lower case letters indicate significant differences among species based on an ANOVA (AN) /Kruskal-Wallis (KW) followed by post-hoc tests with Bonferroni correction (*P* < 0.02).

### Potential germination

*Ailanthus altissima* and scarified *R*. *pseudoacacia* seeds showed a similar and higher potential germination than *F*. *angustifolia* seeds, while non-scarified *R*. *pseudoacacia* seeds showed a similar potential germination to *F*. *angustifolia* seeds (Table 3).

### Potentially recruitable seeds

The expected *PRS* for *R*. *pseudoacacia* would be around twenty times higher when considering that all removed seeds were consumed by birds and germinated at the rate of the scarified seeds. If that was the case, the invaders *A*. *altissima* and *R*. *pseudoacacia* would have the largest number of *PRS*. On the opposite case, if seed consumption prevented germination of *R*. *pseudoacacia* seeds, we would have expected a *PRS* for this species lower than the one of *F*. *angustifolia* (Table 3).

### Seedling abundance

Despite the fact that seedling abundance was almost one order of magnitude higher in *A*. *altissima* than in *F*. *angustifolia*, statistical differences were not found regarding this variable (Table 3). Sexual recruits prevailed over resprouts in *A*. *altissima* plots; this ratio was 1:1 for the native *F*. *angustifolia* and sexual recruits were rare in *R*. *pseudoacacia* plots. However, the intraspecific percentage of sexual recruits was so variable among plots that non-significant differences were found concerning this variable as well (Tables 1 and 3).

## Discussion

In this study we tested several hypotheses related to the success of non-native over native species based on sexual reproductive traits. A greater sexual reproductive success of invasive compared with native species may contribute significantly to their ability to successfully expand and invade new sites. In the following paragraphs we analyse the success of the exotics as compared to the native species through the different stages conducting to a successful sexual reproduction.

### Seed rain

Previous studies characterized invasive plants as having a higher seed production than natives even after accounting for the trade-off between seed number and seed mass [5, 33]. We found a trend of the two invaders to display a higher average seed rain than the native. However, on the basis of the current results (given the high intraspecific variability found for this variable in all three species), we cannot support our first hypothesis. Interspecific comparisons of annual seed rain with wide interannual variation -*Fraxinus* is recognized as a masting genus [70, 71, 72]- would require a long-term study. Unfortunately, little information comprising long-term studies of seed crops for the target species exists, so further studies will be needed to confirm the observed trend through longer time periods.

### Seed infestation and seed removal

Many insects develop inside seeds, being a relevant cause of seed mortality with a significant impact on the reproductive output of plants [73, 74, 75]. These losses did not differ between the invasive and the native species, against predicted by the Enemy Release Hypothesis. However, the fact that they were null in *A*. *altissima*, and significantly larger in the other exotic (*R*. *pseudoacacia*) could be related to the extreme specialization (to one or a few host species) that parasitic lifestyles usually favour. Parasitic lifestyles require complex adaptations to circumvent host defences and sustain life on a single host [76, 77]. On the one hand, *Ailanthus altissima* seeds possess secondary compounds, such as quassinoids, which may have toxic effects [78, 79], whereas the higher protein and starch seed content as well as the lower fiber content in *R*. *pseudoacacia* seeds (see Table 2) might have enhanced its fruit infestation [80, 81, 82]. On the other hand, if a plant species is introduced to a region that contains closely related native congeners, host switching by specialist oligophagous insects to attack the invasive species seems relatively common [83, 84, 24]. This matches with the fact that *Robinia* -despite being an exotic genus in Europe- belongs to a well-represented family in this continent, whose seeds host many parasites [85, 86, 87], whereas *A*. *altissima* has no close taxons in Europe.

The high percentage (35%) of empty seeds displayed by *R*. *pseudoacacia* could be part of a bet-hedging strategy to compensate for its higher seed infestation, diluting the damage caused by oviposition between viable and non-viable (empty) seeds [88, 89, 90]. *Fraxinus angustifolia* also produced nearly 20% of empty seeds. In wind-dispersed species, where animals act as seed predators rather than true dispersers, producing empty seeds is a common strategy to discourage seed predators [91, 92, 93]. *A*. *altissima* might take advantage of its null seed infestation, thus optimizing its seed production instead of devoting resources to produce ‘bait empty seeds’. Accordingly, this species did no produce empty seeds.

We observed that despite their origin (native/exotic) all three species suffered severe seed removal by the local fauna (55–76%), confirming a high reproductive cost at this stage [73, 94]. Against our hypothesis, we found a similar seed removal in the native and in the invaders, again, contradicting the Enemy Release Hypothesis. The putative seed predators, identified from local fauna inventories [95], were rodents (*Apodemus sylvaticus* and *Mus musculus*) and birds (*Serinus serinus*, *Fringilla coelebs*, *Carduelis cannabina*, *Carduelis carduelis* and *Passer domesticus*). Given that all of them are generalist seed consumers, there is no reason to support that they are less likely to remove exotic seeds. For instance, mice tend to consume seeds < 1 cm of diameter, regardless their toxic and nutrient content [96], and granivorous birds select seeds based on their nutrient content, regardless their exotic origin [97, 98]. Indeed the Enemy Release Hypothesis is less supported when local consumers are generalists [99, 100].

### Seed germination

In our experiment, *A*. *altissima* and scarified seeds of *R*. *pseudoacacia* showed higher germination capacity than *F*. *angustifolia*, in accordance with our hypothesis and with previous studies performed outdoors on these species [101]. A high germination rate has been recognized as a reproductive trait promoting invasiveness [5]. The seed dormancy of *R*. *pseudoacacia* may represent a long-term advantage as it allows the plant to form long-lasting soil seed banks (>five years; [102]). This is a bet-hedging strategy also thought to promote invasiveness [5], as it provides the population with a constant source of viable seeds, increasing the chances to find optimal conditions for germinating [103].

### Seedling establishment

Given the similar seed rain and seed removal of the exotics and the native, but the higher seed viability -understood as filled, non- insect-parasited seeds- and germinability of *A*. *altissima* seeds, we would have expected superior seedling abundance in the field for this species as compared to the native. Although the high variability of seedling abundance within each species makes differences non-significant, it is worth mentioning that *A*. *altissima* showed, on average, a seedling abundance which was 8-fold larger than that of the native. We should also mention that most seedlings of *A*. *altissima* corresponded with recently emerged seedlings, a fact not observed in none of the other two species (data not shown). Therefore, there is a possibility that many of the *A*. *altissima* seedlings would have died before reaching their second or third year [104]. Thus, there are evidences suggesting that *A*. *altissima* produces more seedlings than the native, in spite of our failure to significantly detect differences.

On a general basis, it is common to find very low ratios of seedlings per fallen seed (< 0.01) in tree species in the wild [105]. In our study, the number of expected recruitable seeds largely exceeded the seedling abundance found in the field both for the native and the exotic species. These results suggest that the conditions for germination in the study sites were sub-optimal and that seedling emergence in the field is much lower than germination in the laboratory, as found elsewhere [106, 107, 27]. Additionally, there might be a high seedling mortality affecting native and exotic tree species e.g. due to low light (the three species have been reported to be shade-intolerant [72, 108, 109]), to late-spring frosts (seedlings of *F*. *angustifolia* and *A*. *altissima* are frost-sensitive [72, 49]), or/and to summer water stress (seedlings of the exotics have shown high mortality under low soil moisture [101]).

It should be noted that the ratio between seed rain and seedling abundance was especially low for *R*. *pseudoacacia*. There is a chance that the apparent recruitment failure of *R*. *pseudoacacia* may be reflecting an ‘episodic recruitment strategy’, as long-lived tree species do not depend on the short-term success of recruitment [107, 110]. This may be in accordance with the ability of this species to form long-lasting seed banks. Sporadic successful recruitment events may be enough to ensure population persistence [111]. Therefore, the amount of successful recruitment events should be studied in the long-term.

We should also consider that the three studied species may also rely on clonal reproduction to persist in the study site, as many perennial plants do [112, 113]. For instance, in *R*. *pseudoacacia* 95% of the total recruits found had a vegetative origin. This would mean that this species might rely more on clonal growth for maintaining a local dominance. Also, the clones of both *A*. *altissima* and *R*. *pseudoacacia* can survive in the shade, at the expense of reducing their growth rate [114, 115, 116, 45]. Therefore, the study of clonal regeneration patterns would be extremely valuable to predict the fate of these invaded forests.

In conclusion, we cannot support the hypothesis that invaders perform better than native species in terms of sexual reproductive success during the early stages of life-cycle. We found that, regardless their origin, all three species are highly fecund, which allows them to compensate for high removal and/or infestation rates. We did not find evidences to support that native seeds are preferred over exotic seeds by seed consumers. The high intraspecific and inter-annual variability found in many indicators of sexual reproduction success calls for the need to perform long-term studies to better understand the potential of these exotic trees to expand in the future far from the already established populations.

## Supporting Information

S1 Fig Diametric class distributions of *A*. *altissima-*, *R*. *pseudoacacia-* and *F*. *angustifolia-* dominated plots from the middle Henares River riparian forest. DBH- trunk diameter at breast height. Axis y represents the percentage of individuals of each species comprised in every DBH class.
(TIF)

S2 Fig Annual seed rain (*SSN*) expressed as number of seeds per year, hectare and square meter of basal area of reproductive females for the species *A*. *altissima* (invasive), *R*. *pseudoacacia* (invasive) and *F*. *angustifolia* (native) from the middle Henares River riparian forest. (TIF)

S3 Fig Mean percentage of pre-dispersal removed seeds during autumn. (TIF)

S4 Fig Mean percentage of post-dispersal removed seeds during autumn. (TIF)
