# Supplementary figures and images for: Comparing the Sexual Reproductive Success of Two Exotic Trees Invading Spanish Riparian Forests vs. a Native Reference
Source: PLoS One. 2016 Aug 16;11(8):e0160831. doi: 10.1371/journal.pone.0160831 (PMC4987064; doi:10.1371/journal.pone.0160831)

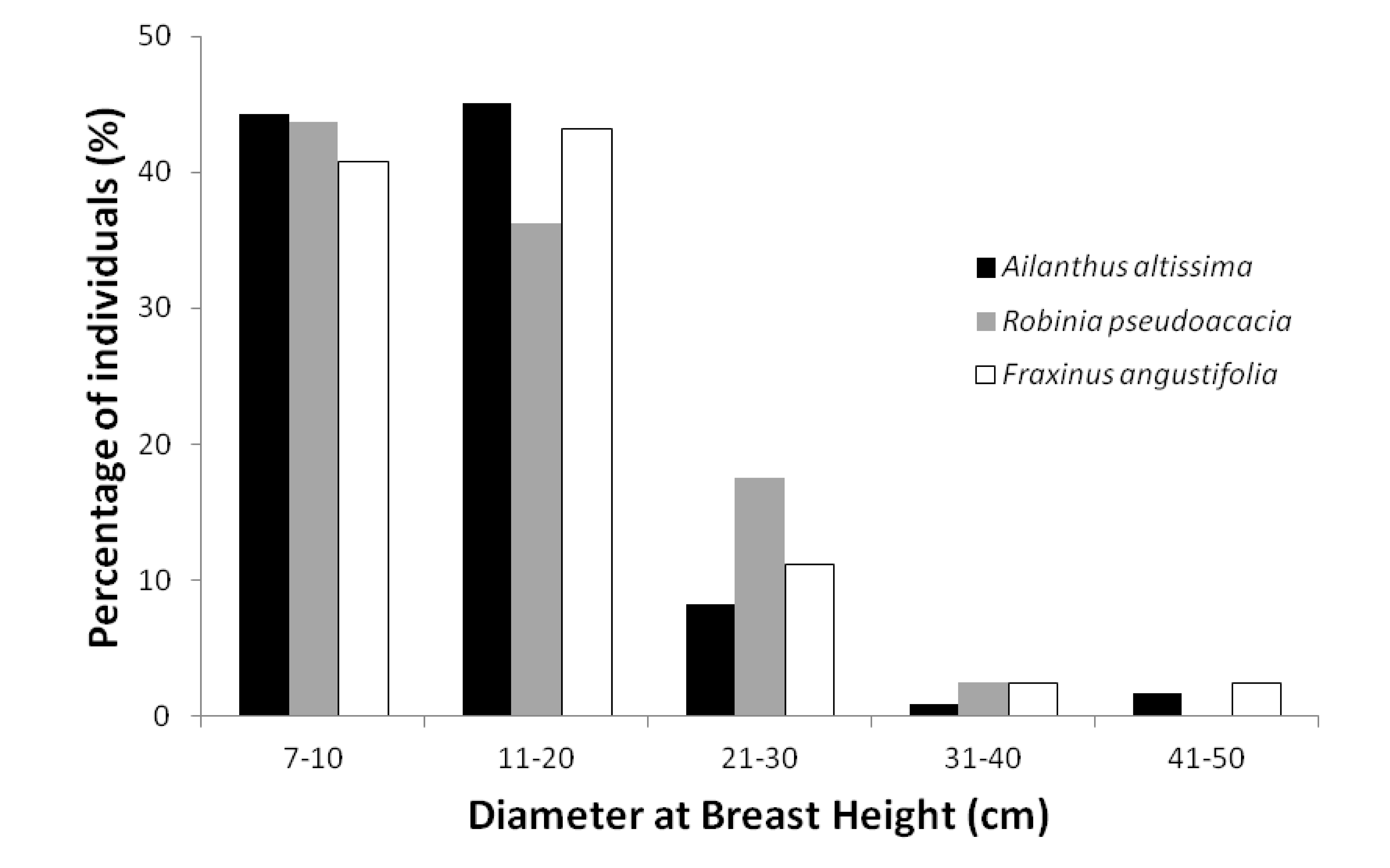

Supplement: S1 Fig — DBH- trunk diameter at breast height. Axis y represents the percentage of individuals of each species comprised in every DBH class. (TIF) [file pone.0160831.s001.tif]

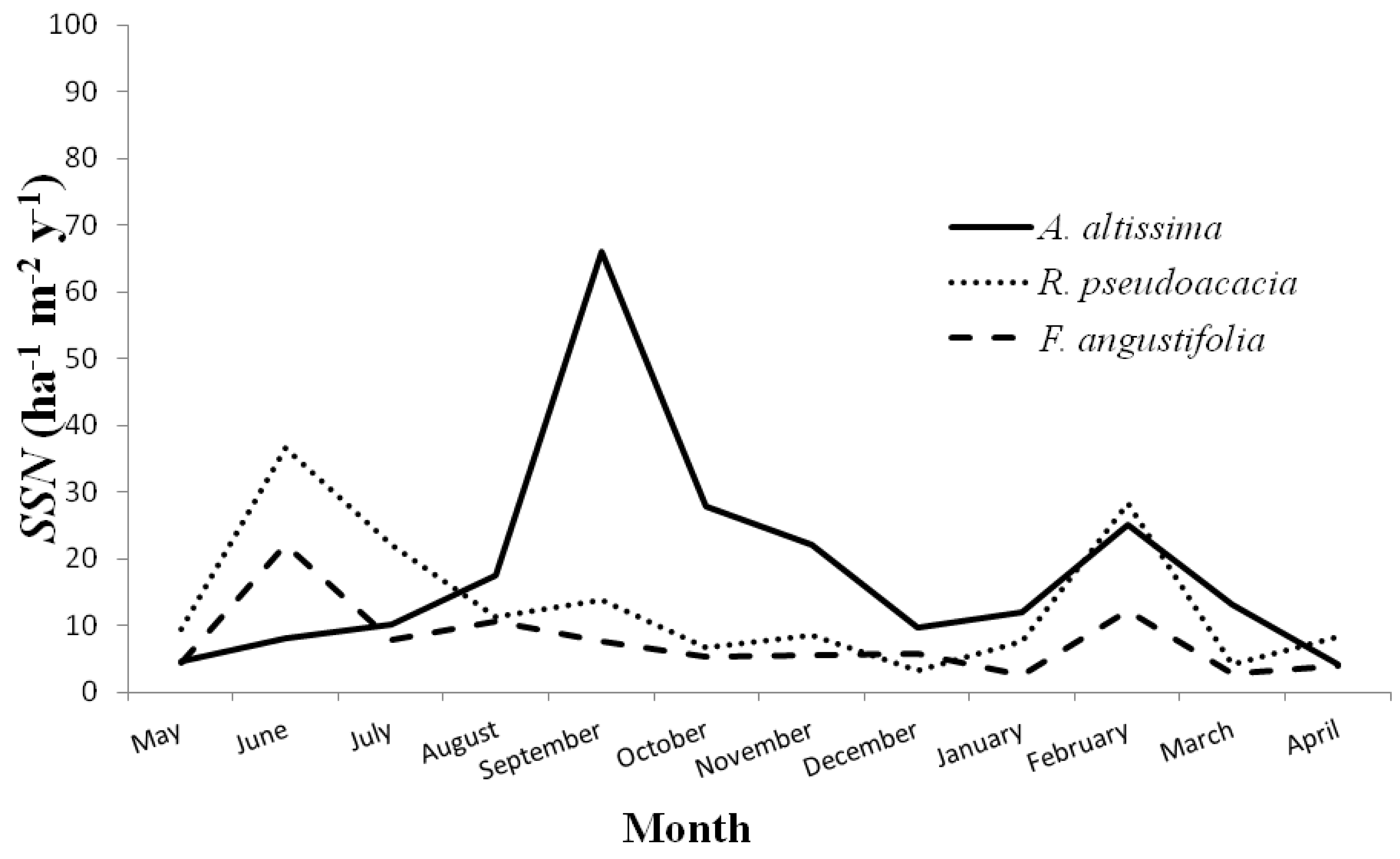

Supplement: S2 Fig — (TIF) [file pone.0160831.s002.tif]

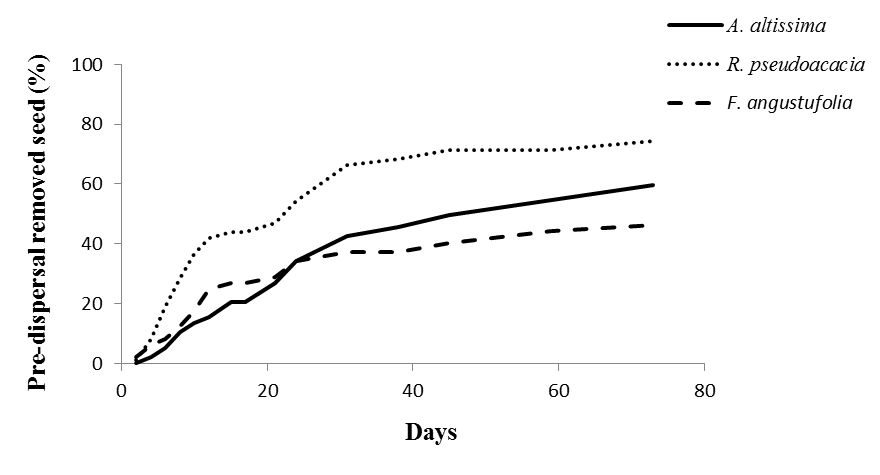

Supplement: S3 Fig — (TIF) [file pone.0160831.s003.tif]

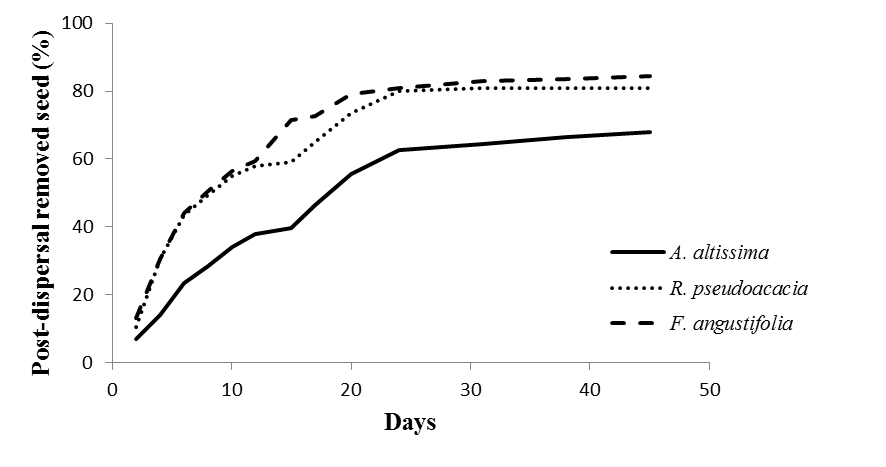

Supplement: S4 Fig — (TIF) [file pone.0160831.s004.tif]
